# Supplementary figures and images for: Phenotypic screening using waveform analysis of synchronized calcium oscillations in primary cortical cultures
Source: PLoS One. 2023 Apr 28;18(4):e0271782. doi: 10.1371/journal.pone.0271782 (PMC10146483; doi:10.1371/journal.pone.0271782)

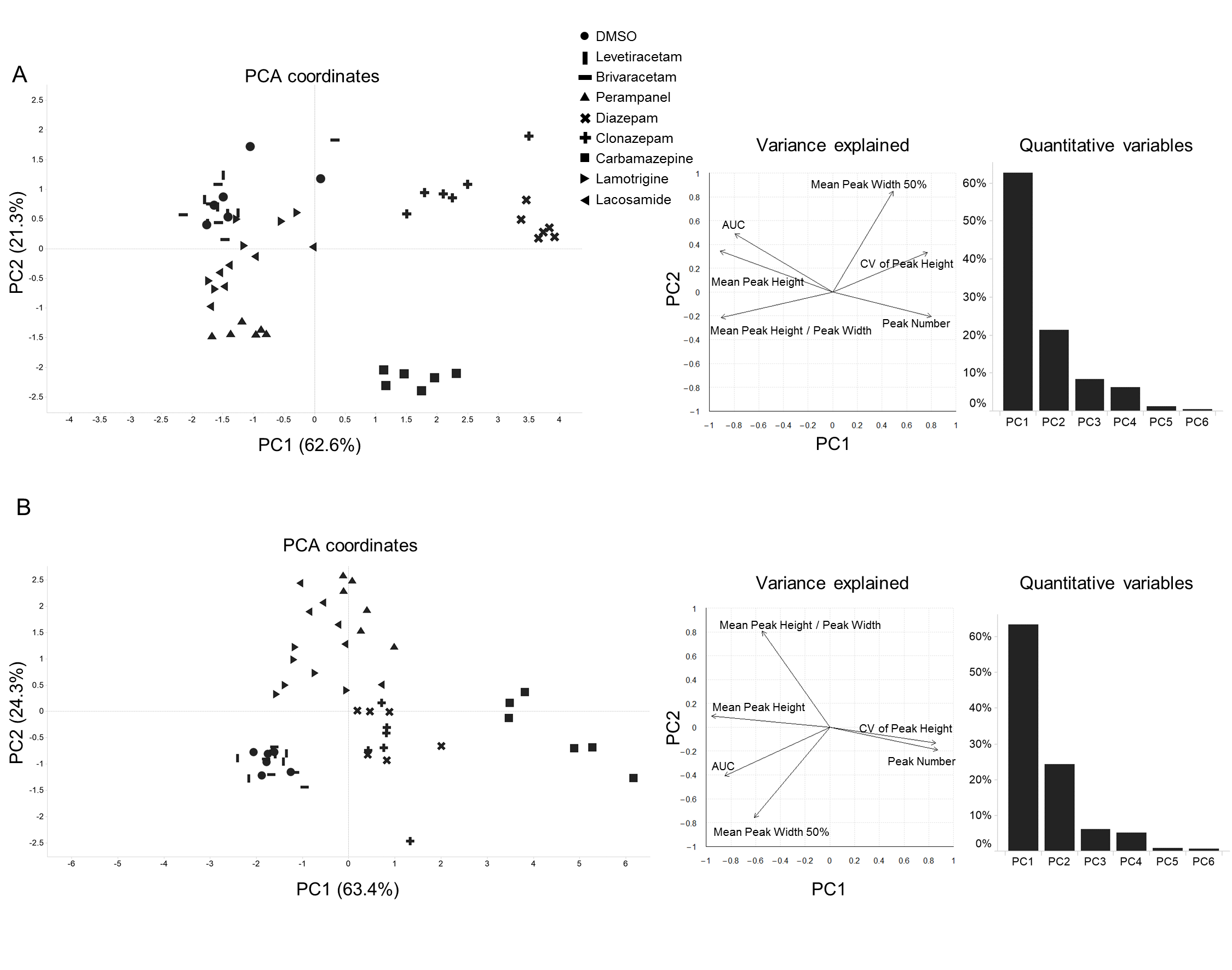

Supplement: S1 Fig — Independent experiments were conducted more than three times. A and B represent the datasets of independent experiments. (TIF) [file pone.0271782.s001.tif]

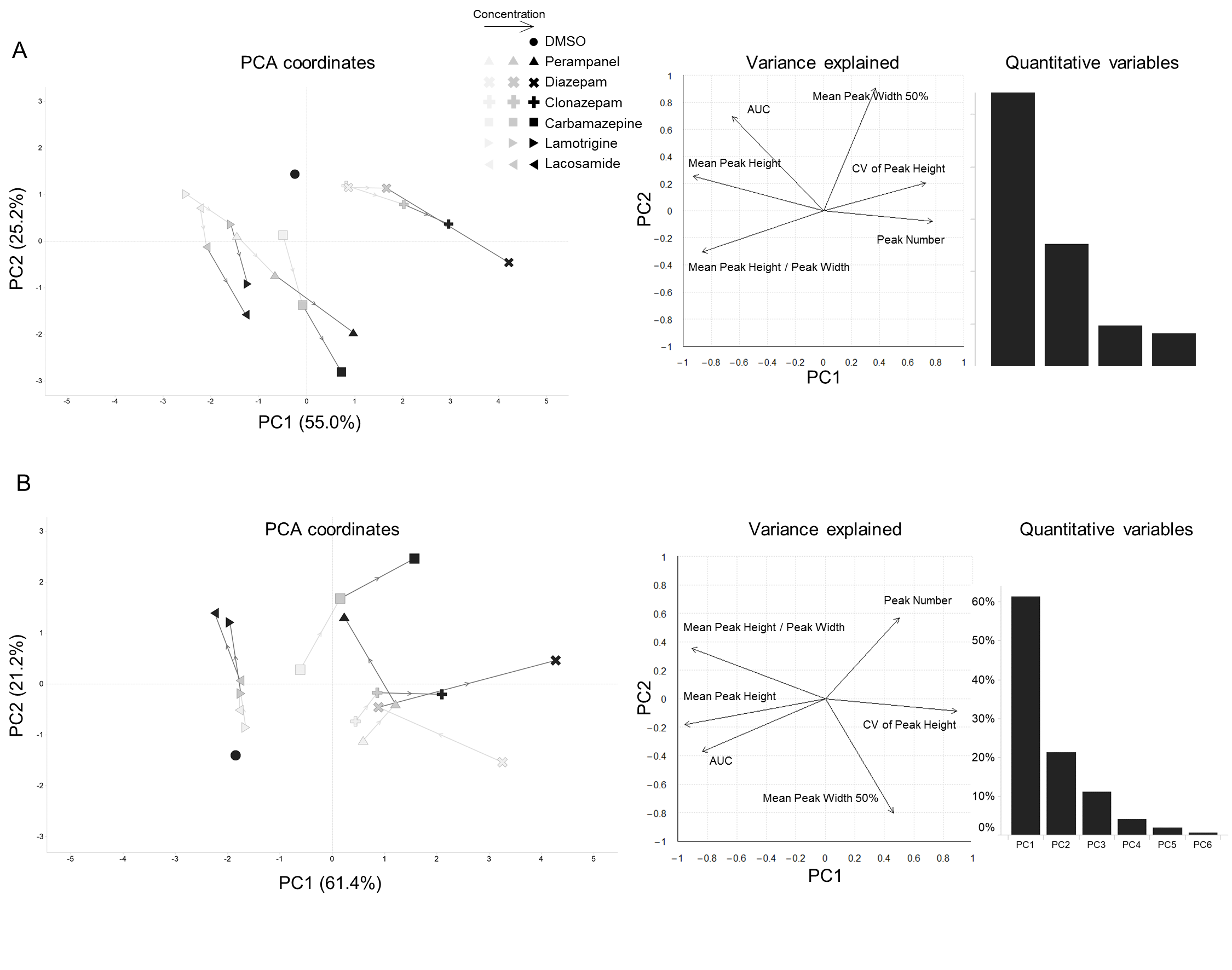

Supplement: S2 Fig — Independent experiments were conducted more than three times. A and B represent the datasets of independent experiments. (TIF) [file pone.0271782.s002.tif]
