# Supplementary material for: Phenotypic screening using waveform analysis of synchronized calcium oscillations in primary cortical cultures
Source: PLoS One. 2023 Apr 28;18(4):e0271782. doi: 10.1371/journal.pone.0271782 (PMC10146483; doi:10.1371/journal.pone.0271782)
Supplement: Attachment — Submitted filename: Response to Reviewers.docx [file pone.0271782.s005.docx]

The pointwise responses to journal requirement and comments of reviewers are also included below and given in red font.

Journal Requirements:

Please review your reference list to ensure that it is complete and correct. If you have cited papers that have been retracted, please include the rationale for doing so in the manuscript text, or remove these references and replace them with relevant current references. Any changes to the reference list should be mentioned in the rebuttal letter that accompanies your revised manuscript. If you need to cite a retracted article, indicate the article’s retracted status in the References list and also include a citation and full reference for the retraction notice.

We thank for indicating our reference style was wrong and incomplete. We amended the style to the Vancouver style. Also, we omitted an un-quoted reference and checked the references again for any omissions.

Additional Editor Comments (if provided):

Kindly address the comments by the reviewers.

[Note: HTML markup is below. Please do not edit.]

Reviewers' comments:

Reviewer's Responses to Questions

Comments to the Author

1. If the authors have adequately addressed your comments raised in a previous round of review and you feel that this manuscript is now acceptable for publication, you may indicate that here to bypass the “Comments to the Author” section, enter your conflict of interest statement in the “Confidential to Editor” section, and submit your "Accept" recommendation.

Reviewer #1: (No Response)

Reviewer #2: All comments have been addressed

Thanks for the careful reading.

2. Is the manuscript technically sound, and do the data support the conclusions?

The manuscript must describe a technically sound piece of scientific research with data that supports the conclusions. Experiments must have been conducted rigorously, with appropriate controls, replication, and sample sizes. The conclusions must be drawn appropriately based on the data presented.

Reviewer #1: Partly

Reviewer #2: (No Response)

Thanks for the careful reading. We added all replicates to our datasets (Figs 3, 5, S1 and S2). Also, we amended the text to mention the variabilities of drug responses caused by sampling dates (L247-250, L275, L316, and L351-355).

3. Has the statistical analysis been performed appropriately and rigorously?

Reviewer #1: Yes

Reviewer #2: (No Response)

We thank all the reviewers for careful and constructive reviews.

4. Have the authors made all data underlying the findings in their manuscript fully available?

The PLOS Data policy requires authors to make all data underlying the findings described in their manuscript fully available without restriction, with rare exception (please refer to the Data Availability Statement in the manuscript PDF file). The data should be provided as part of the manuscript or its supporting information, or deposited to a public repository. For example, in addition to summary statistics, the data points behind means, medians and variance measures should be available. If there are restrictions on publicly sharing data—e.g. participant privacy or use of data from a third party—those must be specified.

Reviewer #1: Yes

Reviewer #2: (No Response)

Thank you for favorable responses.

5. Is the manuscript presented in an intelligible fashion and written in standard English?

PLOS ONE does not copyedit accepted manuscripts, so the language in submitted articles must be clear, correct, and unambiguous. Any typographical or grammatical errors should be corrected at revision, so please note any specific errors here.

Reviewer #1: Yes

Reviewer #2: (No Response)

Thanks for reading our manuscript attentively.

6. Review Comments to the Author

Please use the space provided to explain your answers to the questions above. You may also include additional comments for the author, including concerns about dual publication, research ethics, or publication ethics. (Please upload your review as an attachment if it exceeds 20,000 characters)

Reviewer #1: Sakaguchi and colleagues have significantly improved the manuscipt. Yet, two more points remain to be adressed:

Imaging frequency is still unclear and should be addede to Material and Methods.

I apologized for missing this comment. As advised, we added imaging frequency (27 Hz) to the Materials and methods section (L146).

I would still highly recommend to include the “Other two replicates” into the dataset and not just state (data not shown). To base scientific conclusions or validation of methodology on a single biological replica is not sufficient from my point of view. Especially if individual and “batch-to-batch” (i.e. culture preparation-to-culture preparation) are well described. At least this limitation of the dataset should be mentioned in the manuscript.

We appreciate the enthusiasm on our manuscript. We totally agreed with this reviewer. To comprehend the robustness of our method in spite of variability of drug responses derived from conditions of primary culture, we showed PCA plots, the relative contributions of the principal components and contribution of the different features of all three replicates (Figs 3, 5, S1 and S2). Also, we amend the text to reflect the results of all replicates (L247-250, and L275). Moreover, we declared that there were differences of drug responses depending on sampling dates and we focused on segregation of compounds in this paper because all three replicates prepared individually (L316, and L351-355).

Reviewer #2: The authors responded adequately to my questions and suggestions. I recommend the acceptance of the manuscript for publication.

We appreciate favorable comments on our revision.

7. PLOS authors have the option to publish the peer review history of their article (what does this mean?). If published, this will include your full peer review and any attached files.

If you choose “no”, your identity will remain anonymous but your review may still be made public.

Do you want your identity to be public for this peer review? For information about this choice, including consent withdrawal, please see our Privacy Policy.

Reviewer #1: No

Reviewer #2: No

We agreed to publish the peer review history of this article alongside the article.
